# A cortical multi-layered model and the properties of its internally-generated activity

**DOI:** 10.1186/1471-2202-16-S1-P209

**Published:** 2015-12-18

**Authors:** Rodrigo FO Pena, Renan O Shimoura, Antonio C Roque

**Affiliations:** 1 Departamento de Física, FFCLRP, Universidade de São Paulo, Ribeirão Preto, SP, Brazil

## 

The cerebral cortex displays a rich repertoire of internally-generated dynamic states even in the absence of external stimuli [1]. Most theoretical studies of cortical activity are based on networks of randomly connected units [2] or with architectures artificially built from random networks [3]. In spite of the usefulness of these models, it is also important to have computational models that try to accurately represent cortical network architecture. Recently, Potjans and Diesmann [4] presented a network model of the local cortical microcircuit based on extensive experimental data on the intrinsic circuitry of striate cortex. The model is a full-scale representation of the cortical network under a surface area of 1 mm^2 ^of striate cortex (~80,000 neurons) and contains two cell types (excitatory and inhibitory) distributed over four layers: L2/3, L4, L5, and L6. The cells are modeled as current-based leaky integrate-and-fire neurons with exponential synaptic currents. In this work, we used the connectivity map of the Potjans and Diesmann model [4] to construct a cortical model with 4,000 neurons (i.e. with the number of cells reduced by a factor of 20 in comparison with the Potjans and Diesmann model). Cells were described by the Izhikevich model [5] with parameters adjusted so that excitatory neurons were of the regular spiking (RS) type, 50% of the inhibitory neurons were of the fast spiking (FS) type and the other 50% of the inhibitory neurons were of the low-threshold spiking (LTS) type. Synapses were modeled as conductance-based with exponentially decaying conductances (we used the same synaptic parameters as in [3]). Instantaneous excitatory/inhibitory synaptic increments were denoted by *g*_ex_/*g*_in_. Brief (10 ms) but strong direct current pulses were applied to 15% of L4 excitatory cells to stimulate the network and, after stimulus removal, we kept the simulation running until *T*_sim _= 3000 ms. We performed this experiment for 10 different initial conditions to randomize the construction of the network as well as for at least 100 different combinations of *g*_ex_/*g*_in _in the range *g*_ex _= [0, 0.1], *g*_in _= 0[1]. The measurements taken were the lifetime of network activity, the activity of the network and the coefficients of variation of the interspike intervals of network neurons. In addition, we performed the same experiments with L4 isolated and in all possible combinations (in pairs or triplets) with the other layers, and with 100% of inhibitory cells of the FS type.

The major results of our simulations are: (1) For networks made of RS and FS cell types, long-lived network activity was observed for combinations of *g*_ex_/*g*_in _in the region of highest values of both of them. These states displayed irregular neuronal firing. For other combinations of *g*_ex_/*g*_in _the network activity decayed rapidly after a short transient; (ii) Introduction of LTS neurons increased the region of *g*_ex_/*g*_in _combinations that generated long-lived activity and reduced the average network firing rate; (iii) Different combinations of layers favored more or less the occurrence of long-lived activity. L4 alone, L4-L5 and L4-L5-L6 could not sustain long-lived activity while L4-L6 and L23-L4-L6 displayed long-lived activity for larger regions of *g*_ex_/*g*_in _combinations.
